# Kinetic inhibition effect of Type I and III antifreeze proteins on unidirectional tetrahydrofuran hydrate crystal growth

**DOI:** 10.1039/c9ra00627c

**Published:** 2019-04-11

**Authors:** Michihiro Muraoka, Michika Ohtake, Yoshitaka Yamamoto

**Affiliations:** Research Institute of Energy Frontier, National Institute of Advanced Industrial Science and Technology (AIST) Onogawa Tsukuba Ibaraki 305-8569 Japan m-muraoka@aist.go.jp +81 29 861 8765 +81 29 861 2841

## Abstract

Experiments were performed to evaluate the kinetic inhibition effect of Type I and Type III antifreeze proteins (AFPs), polyvinyl alcohol (PVA) and polyvinylpyrrolidone (PVP) on the growth of tetrahydrofuran (THF) clathrate hydrate crystals using the unidirectional growth technique. The crystal growth interface shifted under an applied temperature gradient over a given time. Type I AFP was the most effective kinetic inhibitor, followed by PVP, Type III AFP and PVA. The morphology of the THF hydrate was affected significantly by the concentration of Type I AFP. At low concentrations of Type I AFP, a large hexagonal plate-like crystal formed. At high concentrations, the growth interface became relatively flat. Conversely, at low concentrations of Type III AFP, the morphology of the THF hydrate crystal was unaffected by the additive.

## Abstract 2

Experiments were performed to evaluate the kinetic inhibition effect of Type I and Type III antifreeze proteins (AFPs) on the growth of tetrahydrofuran (THF) clathrate hydrate crystals using the unidirectional growth technique.

## Graphical abstract

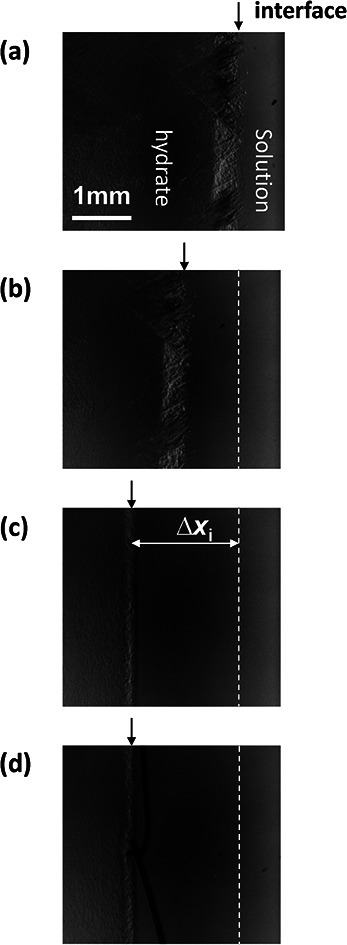

## Introduction

Gas hydrates are ice-like crystalline solids that trap guest molecules of gas within the cells formed by the hydrogen bonds of water.^1^

The formation of natural gas hydrate plugs during petroleum extraction and transportation has been a serious problem for many years.^2^ Thermodynamic inhibitors (THIs) such as methanol are often used to inhibit the formation of hydrates in pipelines. Such inhibitors shift the equilibrium state of the gas hydrates to a higher pressure and lower temperature. However, high doping is necessary to prevent hydrate formation. Novel inhibitors called kinetic hydrate inhibitors (KHIs) have been developed, requiring much less doping to effectively inhibit hydrate formation. KHIs primarily act by delaying the nucleation and slowing the growth rate of the hydrate.^3^ Evaluating and comparing the performance of KHIs and THIs is very difficult.

Lederhos *et al.* evaluated kinetic inhibitors among 1500 chemicals.^4^ They used 12 test tubes (rocking cell) filled with a tetrahydrofuran (THF)/water solution, and each tube contained a stainless-steel ball. They screened KHIs by monitoring the time period for which the ball movement was blocked by the formed hydrate.

Larsen *et al.* evaluated KHIs using single crystals of THF and ethylene oxide (EO) hydrate.^5^ THF and EO hydrate have structure II and I, respectively. Authors measured the minimum concentration required to obtain complete inhibition of crystal growth *vs.* the degree of super cooling. They also reported that the morphology of THF hydrate changed from octahedra to 2-dimensional hexagonal plates owing to the KHIs effect.

Anderson *et al.* developed a procedure named the crystal growth inhibition (CGI) method for evaluating KHI performance.^6^ In the CGI method, KHIs are tested over several cycles of hydrate growth and melting cycles with the repeated system cooling and heating being maintained at a constant rate. The rocking-cell system is also used for the efficient evaluation of KHI performance. Chua *et al.* used five high-pressure steel rocking cells.^7^

Successful inhibitors were found by trial and error.^4^ On the contrary, some of the current KHI designs were inspired by research on antifreeze proteins (AFPs)^8^ that suppress the growth rate of ice crystals and change the crystal morphology. The inhibiting action of AFPs on ice recrystallisation and growth helps living organisms survive in subfreezing environments.^9^

Type I AFP was initially discovered in the blood plasma of a fish, the winter flounder;^10^ it has a simple α-helical structure.^11^ At low concentrations, it adsorbs on the bipyramidal plane surfaces of ice crystals,^12^ whereas at high concentrations, it adsorbs on all orientation surfaces of ice crystals, including on the basal planes.

Type III AFP, a globular protein, was first discovered in an ocean pout (Zoarcidae or eelpouts);^13,14^ it adsorbs on the prism and pyramidal planes of ice crystals.^15^

Zeng *et al.* tested the effects of Type I AFP and another AFP Type extracted from insects to inhibit the growth of (THF) hydrate. The results showed that both AFPs affect the morphology and inhibit nucleation of THF hydrate crystal.^16^ In addition, they showed that both AFPs eliminate the memory effect.

Zeng *et al.* tested the effects of Type I AFP to inhibit the growth of methane hydrate and propane hydrate.^17^ Both nucleation and crystal growth were inhibited using AFP.

Daraboina *et al.* studied the inhibition effects of TYPE III AFP and PVP for gas hydrate.^18–20^ Their results showed that AFP III and PVP delayed the onset of hydrate nucleation.^18^ Their results also showed that AFP I was a more effective than either PVP or TYPE III AFP.^19^

Polyvinyl alcohol (PVA) is a water-soluble polymer with hydroxyl groups exhibiting a multiple-stranded, helical structure when dissolved in water. Inada *et al.* and Wang *et al.* found that PVA inhibits the growth of ice.^21,22^ Budke and Koop and Inada *et al.* reported that PVA inhibits the recrystallisation of ice.^23,24^ Du *et al.* found that the adhesion force of THF hydrate particle is weakened by the addition of PVA.^25^

DuQuesnay *et al.* developed a novel gas hydrate reactor that enables the observation of the morphology of gas hydrate formed under a temperature gradient.^26^ Udegbuman *et al.*^27^ used the same reactor to evaluate AFPs from fish, beetle, and grass. They reported the relationship between film hydrate velocity and super cooling.

In most of the KHI performance evaluation experiments, gas hydrate was formed by stirring gas and water in a high-pressure reactor, or THF hydrate forms in a rocking cell. These types of experiments cannot separate the inhibition effect of growth and nucleation. The data exclusively focused on only evaluating the growth inhibition effect, which is scarce. We consider that simplified experimental data is necessary to clarify the KHI mechanism.

In a previous publication, we proposed a simple method for evaluating KHI performance with a unidirectional growth apparatus.^28^ We measured the degree of supercooling while controlling the rate of crystal growth to evaluate the antifreeze performance of polyvinylpyrrolidone (PVP). This technique eliminates nucleation and solely evaluates growth inhibition effect. It allows for easy quantification of the inhibition performance and the observation of interfacial phenomena during the hydrate crystal growth.

In this study, we evaluate the kinetic inhibition performance of Type I AFP, Type III AFP, PVA and PVP on the growth of THF clathrate hydrate crystals using the unidirectional growth technique. In addition, we analyse the differences in the inhibitory action of Type I and Type III AFP.

## Experimental methods

A stoichiometric THF–water solution (1 : 17) prepared from dehydrated stabiliser-free THF (99.5 wt% purity, Kanto Chemical Co., Japan) and ultrapure water (18.2 MΩ × cm resistivity) was used for the tests. High-purity Type I AFP extracted from the winter flounder (average molecular weight = 3200, Nichirei Co., Japan), high-purity Type III AFP from the ocean pout or eelpout (average molecular weight = 6700, Nichirei Co., Japan), PVA (98% purity, average molecular weight = 16 000, Kanto Chemical Co., Japan) and PVP (K-90, average molecular weight = 1 200 000, Junsei Chemical Co. Ltd., Japan) were added to the THF–water solution for testing.

The present study employed the same experimental methods that we reported previously;^28^ we summarised the setup as follows.

The sample cell comprised two glass plates (26 × 76 × 1 mm) and rubber spacers. Two thermocouples (Type T, 01-T, Ninomiya Electric Wire Co. Ltd., Japan) were inserted between the rubber spacer and the glass plate. The calibrated thermocouples are accurate within 0.2 °C. The measurement positions of the thermocouples were ∼5 mm (no. 1) and 15 mm (no. 2) from the right inner edge of the cell.

The unidirectional growth apparatus generates a constant temperature gradient *G* across the sample cell *via* a set of cold and hot copper blocks. The sample cell was placed on the cold and hot blocks. The hot block was held at a temperature above the equilibrium temperature *T*_eq_ while the cold block was held at a temperature below *T*_eq_. The thermocouple measurements were recorded by a data logger (midi Logger GL840, Graphtec Co., Japan). The nucleation of hydrate crystals was initiated by cooling the colder side of the capillary using a cold spray (134a QREI, QRA -S481, Sunhayato Co., Japan). Then, an initial growth interface of an extra 0.3 mm was formed to ensure a flat interface. Next, excess hydrates were melted by moving the sample cell slightly towards the hot block, the cell was kept still for 2 h. After these preparations, the sample cell was moved using a pulse motor at constant velocity (*V*) towards the colder block. The cell was moved a total distance of 25 mm towards the colder block in each experiment.

The optical system comprised an optical zoom lens (VZM 600I, Edmund optics) and a CCD camera (EO-1312M, Edmund optics). The CCD camera images were recorded on a personal computer running on the Windows operating system (Windows 8).

The KHI concentration (*c*) and *V* were the independent variables in this study.

## Results and discussion

**Fig. 1:**
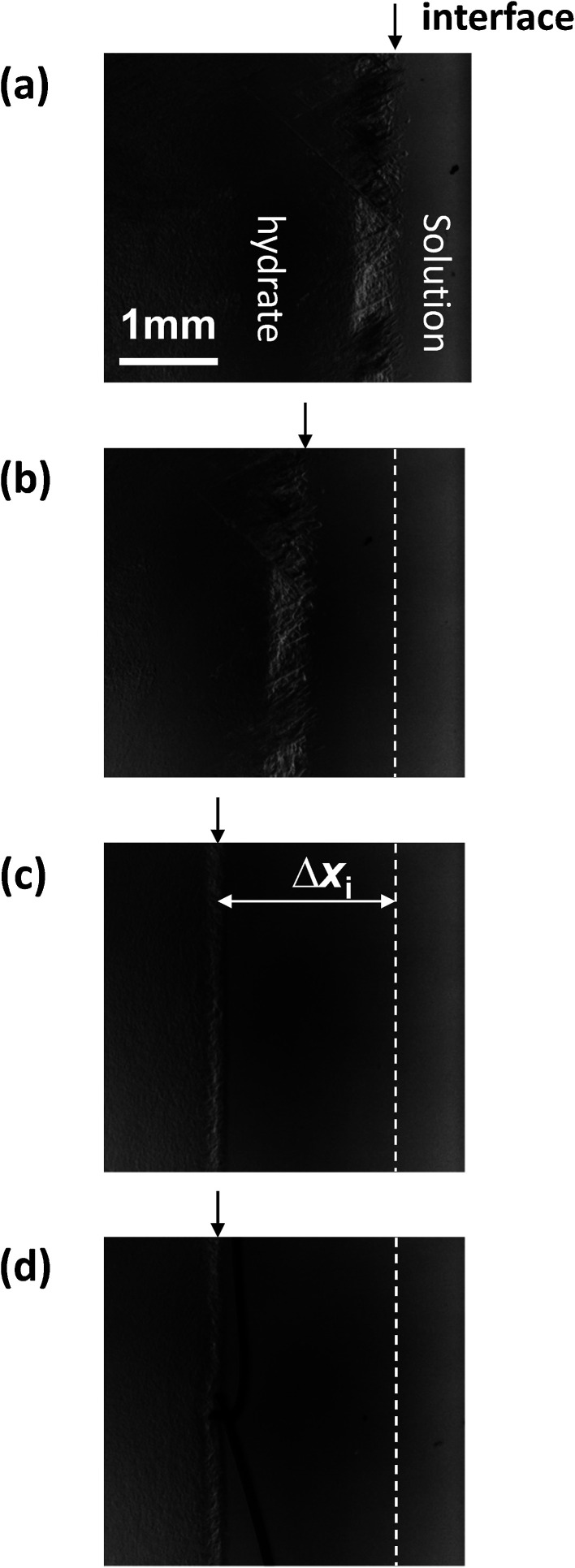
Sequential images of the growth interface of THF hydrate with the additive Type I AFP at a concentration of *c* = 0.5 wt% and *V* = 1 μm s^−1^. The growth times are (a) *t* = 0 min, (b) *t* = 13 min, (c) *t* = 125 min and (d) *t* = 231 min.

Fig. 1 shows sequential images of the hydrate growth interface of THF aqueous solution (THF–17H_2_O) with Type I AFP (*c* = 0.5 wt% and *V* = 1 μm s^−1^). The time elapsed from the start of the crystal growth shown in Fig. 1 are (a) *t* = 0 min, (b) *t* = 13 min, (c) *t* = 125 min, and (d) *t* = 231 min. The single arrow in each figure indicates the position of the crystal growth interface. On observing the details, the microscopic interface shape comprises many fine needles. From *t* = 0 to *t* = 13 min, the growth interface gradually shifted toward the cold block as it was moved through the temperature gradient. After 125 min [Fig. 1(c)], the interface position was constant, indicating the system was steady state. At *t* = 231 min [Fig. 1(d)], the growth interface reached the thermocouple tip (no. 2), located at 15 mm from the right edge of the cell. The length of double-headed arrow Δ*x*_i_(*t*) [Fig. 1(c)] shows the distance between the crystal growth interface position at a certain time and its position at the beginning of the growth (*t* = 0). The values of Δ*x*_i_ were obtained by averaging 10 measurements at different positions along the interface. “Δ*x*_i_” indicates the excess of supercooling caused by KHI doping, relative to the supercooling that occurs in a simple THF aqueous solution. From measurements of Δ*x*_i_(*t*) at steady state, we can calculate the supercooling due to the KHI additive (Δ*T*_i_).^28^

In our previous study, we calculated the temperatures at each position of the sample cell based on the temperature gradient, and the distance from each thermo-block. Δ*T*_i_ was calculated using the simple formula in (1):

1 Δ*T*_i_ = *G*_ave_ Δ*x*_i_,

where *G*_ave_ was the linear temperature gradient and was calculated as *G*_ave_ = (*G*_1_ + *G*_2_)/2, where *G*_1_ and *G*_2_ were the temperature gradients recorded by thermocouples no. 1 and 2, respectively. In the present experiments, *G* is approximately 1.9 °C mm^−1^ in all experimental runs. To check the accuracy of this method, we compare the values of Δ*T*_i_ estimated from Formula (1) and those measured directly from the thermocouple tips inside the cell.

In the cases of THF aqueous solutions containing PVP or PVA or TYPE III AFP, the calculated and directly measured crystal growth interface temperatures agree well within the error of the measurements. However, in the case of AFP Type I solutions, the two interface temperatures do not agree.

**Fig. 2:**
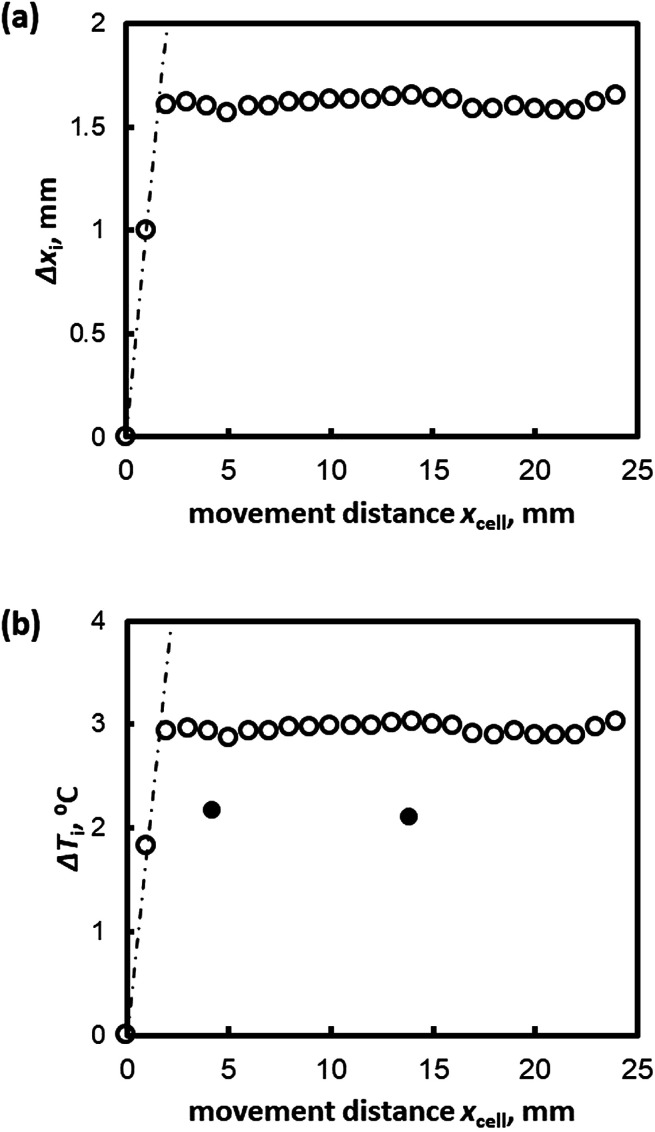
(a) Δ*x*_i_ and (b) Δ*T*_i_ plotted against the distance moved by the sample cell, *x*_cell_(*t*), for *V* = 1 μm s^−1^ for Type I AFP *c* = 1.0 wt%.

Fig. 2(a) shows the relation between Δ*x*_i_(*t*) and *x*_cell_(*t*) (the distance the sample cell moved) for Type I AFP (*V* = 1 μm s^−1^, *c* = 0.5 wt%); the dotted line represents the hypothetical line of Δ*x*_i_ = *x*_cell_. If the position of the growth interface relative to the cell would remain constant (*i.e.* the hydrate does not grow after *t* = 0), the interface behavior would develop according to the linear relationship shown by the dotted line. However, the results show that as *x*_cell_ increases, Δ*x*_i_ also increases within the first 5 mm of the cell motion. Beyond *x*_cell_ = 5 mm, Δ*x*_i_ remains almost constant in all cases. Fig. 2(b) plots the degree of supercooling at the growth interface, Δ*T*_i_, against *x*_cell_.

The white and black circles in Fig. 2(b) indicate the directly measured degree of supercooling and the values as calculated from Formula (1), respectively. The calculated values of Δ*T*_i_ and the measurements at the thermocouple tip differ; Formula (1) overestimates Δ*T*_i_ in the case of Type I AFP.

Celik *et al.* showed that ice can be superheated in AFP solutions under isothermal conditions.^29^ Knight and Devries developed a schematic model that explains how AFP bound to the ice-crystal surface allows superheating.^30^

The observed disagreement between the calculated values of Δ*T*_i_ from Formula (1) and the measured values may be explained by superheating at the initial growth interface. The initial interface is prepared by melting 0.3 mm of hydrate. Normally, the temperature of the initial interface is at the melting temperature of the simple THF aqueous solution (4.4 °C). However, the initial interface is in contact with the AFP solution in this case, which should cause superheating of the THF hydrate crystals.

To determine the growth interface temperature, the formula Δ*T* = *G*Δ*x* is normally implemented. However, since AFP is used in the experiment, Formula (2) should be applied instead.

2 Δ*T* = *G*Δ*x* − Δ*T*_s_,

where Δ*T*_s_ is the degree of superheating of the initial interface at *t* = 0 min. Δ*T*_s_ may be influenced by the melting time, the type of KHI added and the KHI concentration.

An exact comparison of calculated values and direct temperature measurements also reveals that *V* slightly affects the *G* of the sample cell. Therefore, to be more precise, Formula (2) should be modified to account for the motion of the sample cell.

3 Δ*T* = *G*(Δ*x* − *x*_T_) − Δ*T*_s_

In Formula (3), *x*_T_ is the distance the isotherm is shifted towards the cold side because of the cell motion. *x*_T_ should increase with increasing *V*. To evaluate the interface temperature using Formula (3), *x*_T_ and Δ*T*_s_ must be determined empirically for each KHI. Formula (1) can only be applied under experimental conditions with lower range values of *V* and KHI additives with a small superheating effect.

In the case where no additive solution is present (THF–water solution), Δ*T*_s_ = 0. Formula (3) becomes Formula (4).4
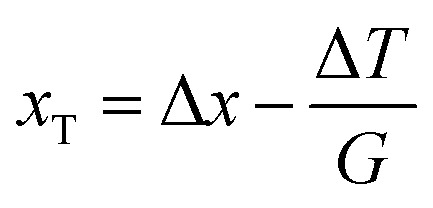


**Fig. 3:**
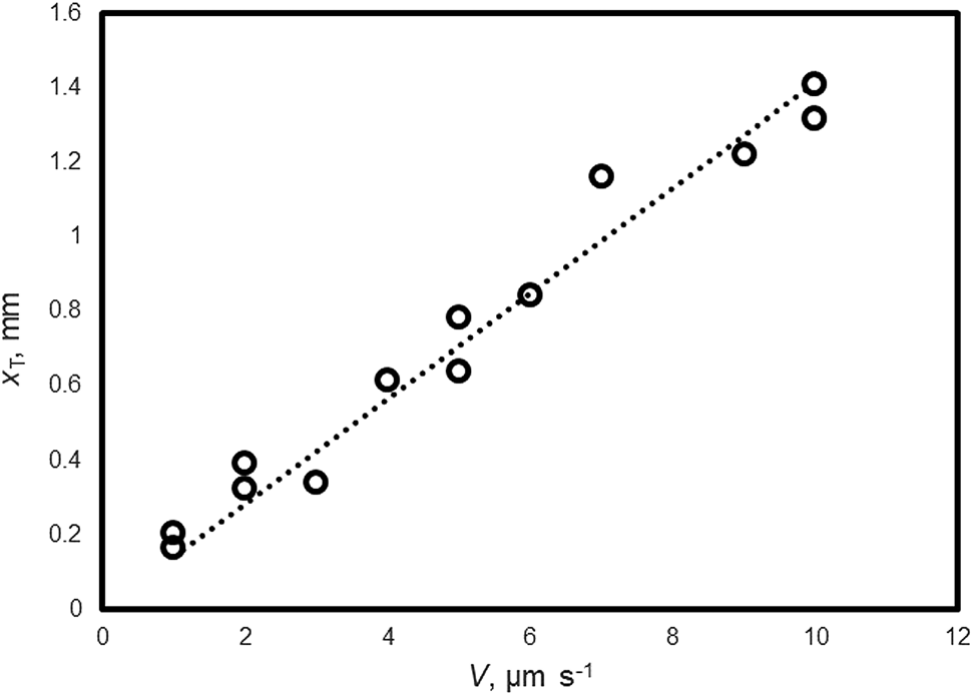
The relationship between *V* and *x*_T_ of THF hydrate (no additive).

Fig. 3 shows the relationship between *x*_T_ and *V* of the THF hydrate (no additive). *x*_T_ is obtained by Formula (4) and the measured values of Δ*x*, Δ*T* and *G*. By carrying out a linear fit, *x*_T_ = 0.14 *V* is obtained.

**Fig. 4:**
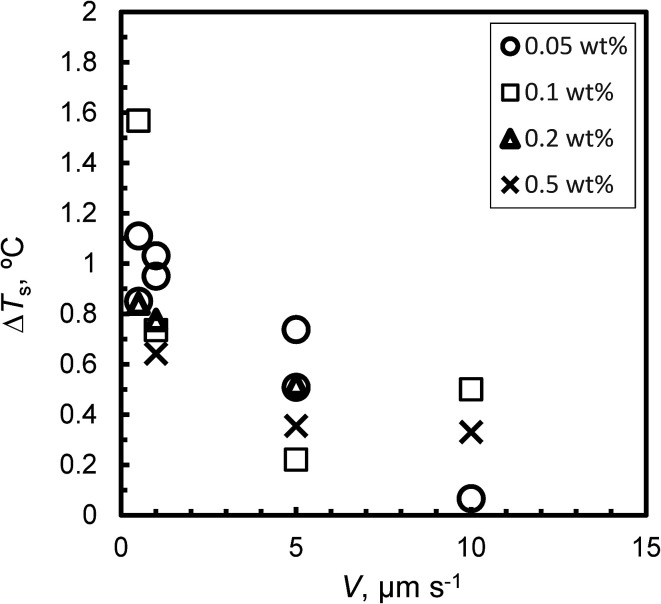
The relationship between *V* and degree of superheating, Δ*T*_s_, of the initial interface.

Fig. 4 shows the relationship between Δ*T*_s_ and *V* for Type I AFP; Δ*T*_s_ is not dependent on *c*. For the same experimental condition as for Δ*T*_s_ = 0, a variation of approximately 0.3 °C is found when Type I AFP is included. Therefore, the temperature of the growth interface is measured directly using a thermocouple to avoid this error.

**Fig. 5:**
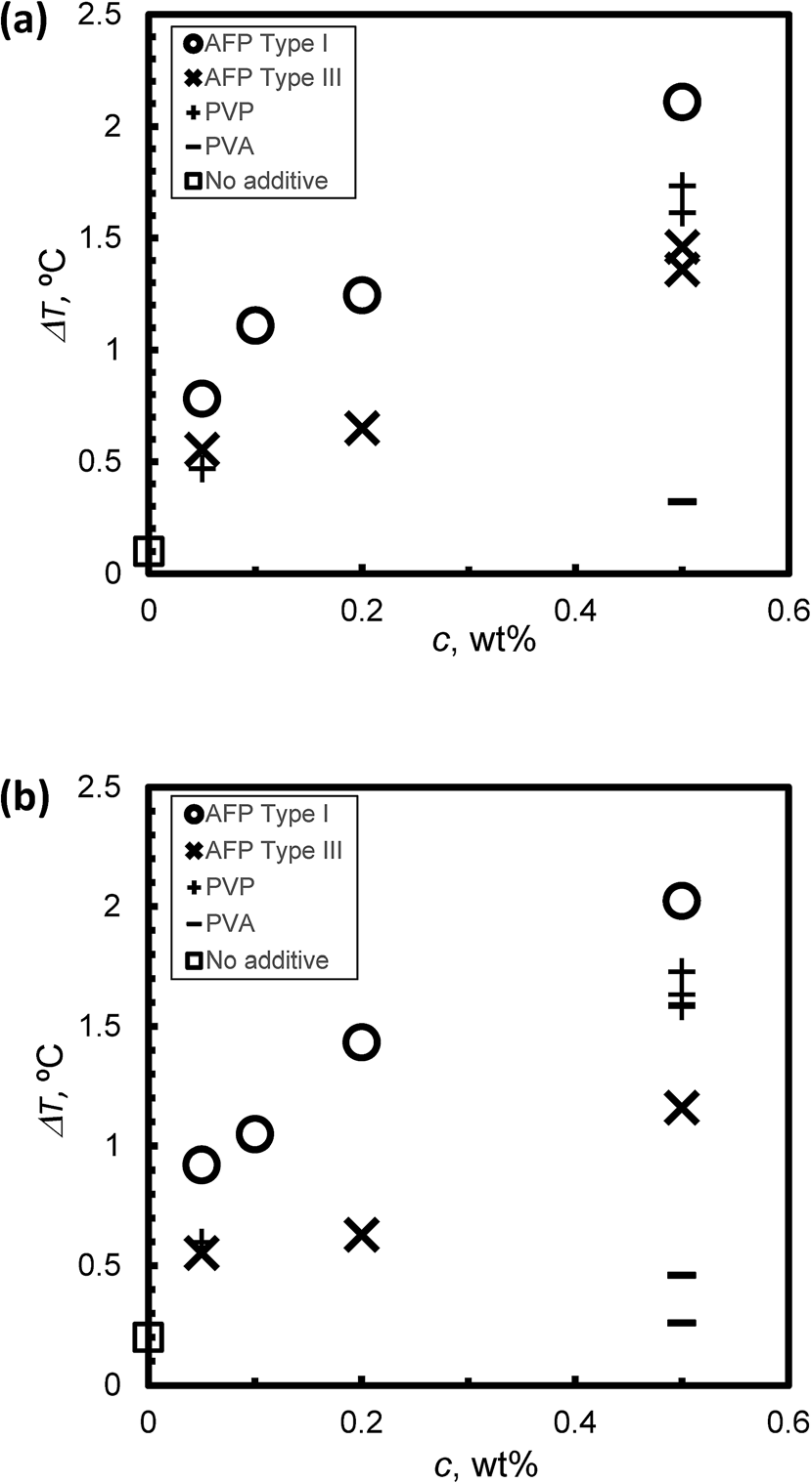
The relationship between KHI concentration, *c* and the degree of super cooling, Δ*T*, under steady state conditions at (a) *V* = 1 μm s^−1^ and (b) *V* = 5 μm s^−1^.

Fig. 5a and b summarize the relation between the KHI concentration *c* and the degree of supercooling Δ*T* under steady state conditions at (a) *V* = 1 μm s^−1^ and (b) *V* = 5 μm s^−1^, respectively. Δ*T* increased along with *c* in all cases. In the case of *c* = 0.5 wt%, the Δ*T* of Type I AFP exhibits the best performance. The inhibition performance of PVP is second best followed by Type III AFP; PVA performs weakly. The result of PVA is consistent with that in ref. 5; this study reported that PVA is not an inhibitor.

**Fig. 6:**
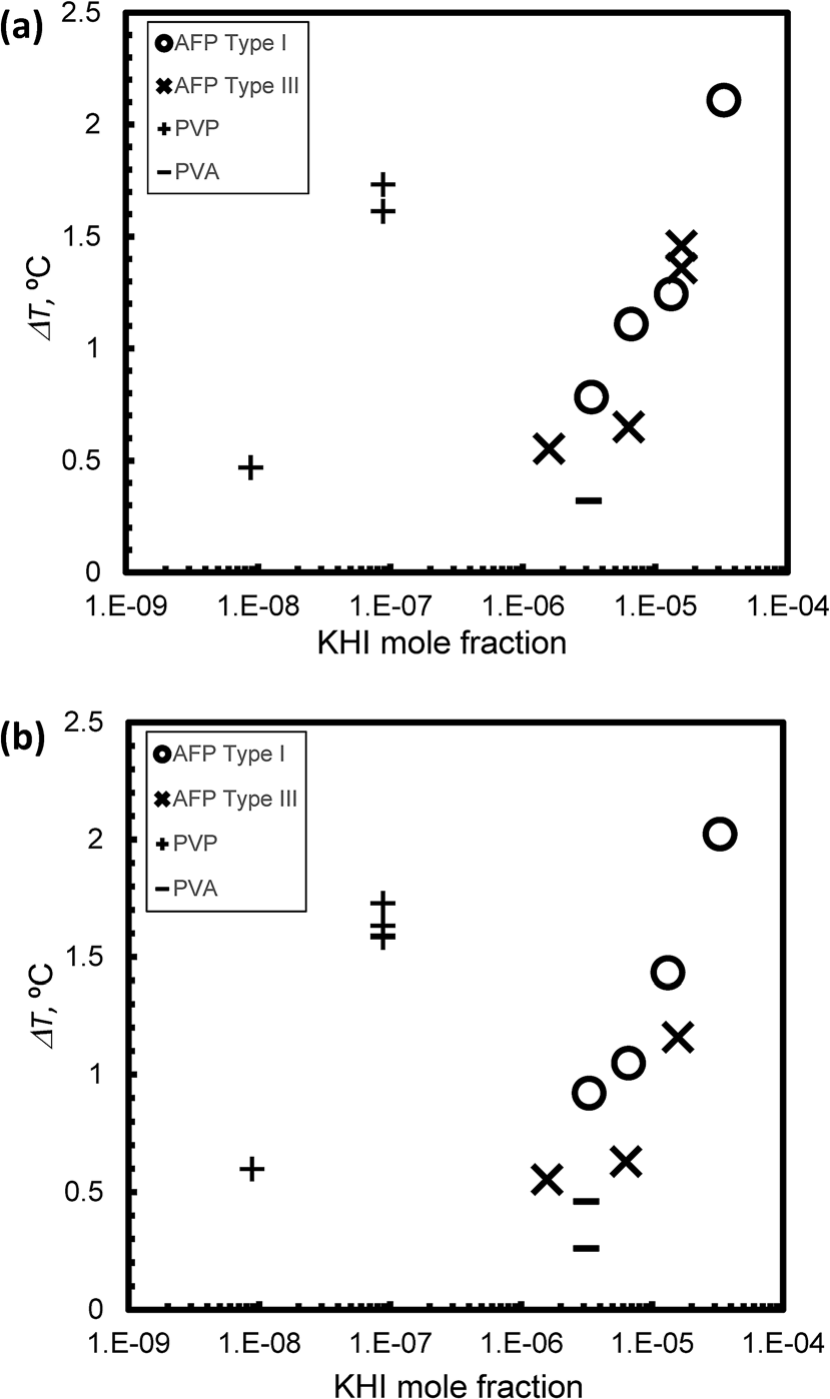
The relationship between KHI mole fraction and degree of super cooling, Δ*T*, under steady state conditions at (a) *V* = 1 μm s^−1^ and (b) *V* = 5 μm s^−1^.

Fig. 6(a) and (b) summarize the relationship between KHI mole fraction and degree of supercooling, Δ*T*, under steady state conditions at *V* = 1 μm s^−1^ and *V* = 5 μm s^−1^, respectively. The KHI mole fraction of PVP was less than 2 orders of magnitude as compared to AFPs. This is caused by the molecular weight of PVP that is larger than AFPs. The KHI mole fraction of PVP is extremely low as compared to AFPs; however, the Δ*T* of PVP is of the same order magnitude as Δ*T* of the AFPs. This result suggests that a single molecule of PVP has multiple adsorption sites on the hydrate surface. Thus, to clarify the KHI inhibition mechanism, it is important to determine the number of adsorption sites of a single KHI molecule.

The KHI inhibitor performance is summarized by the following ranking: Type I AFP > PVP > Type III AFP > PVA. The result of TYPE I AFP is consistent with that in ref. 19.

**Fig. 7:**
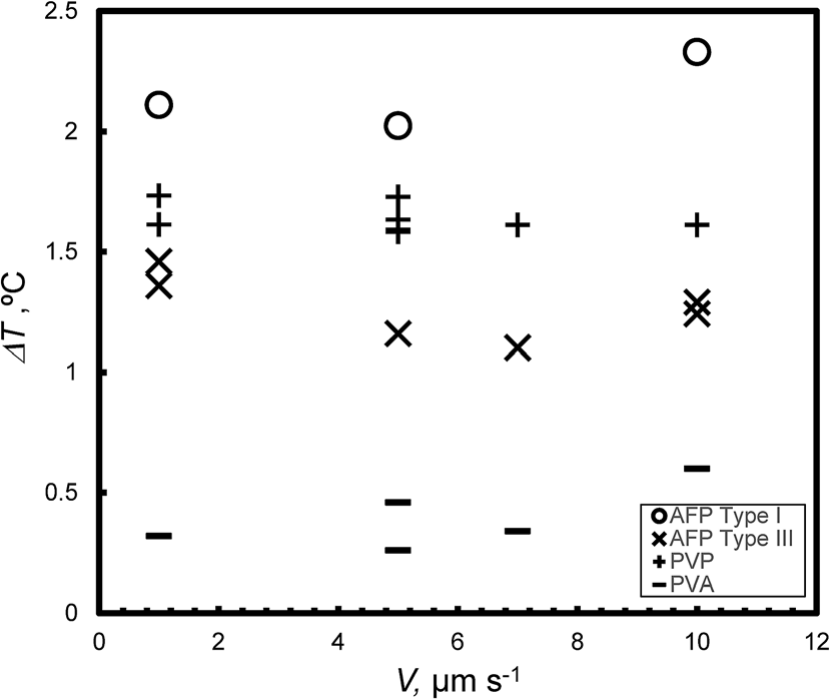
The relationship between *V* and the degree of super cooling, Δ*T*, under steady state conditions at *c* = 0.5 wt%.

Fig. 7 presents the weak dependence of the degree of supercooling (Δ*T*) on *V* under steady state conditions at *c* = 0.5 wt%; for each KHI, the Δ*T* is similar for different values of *V*.

**Fig. 8:**
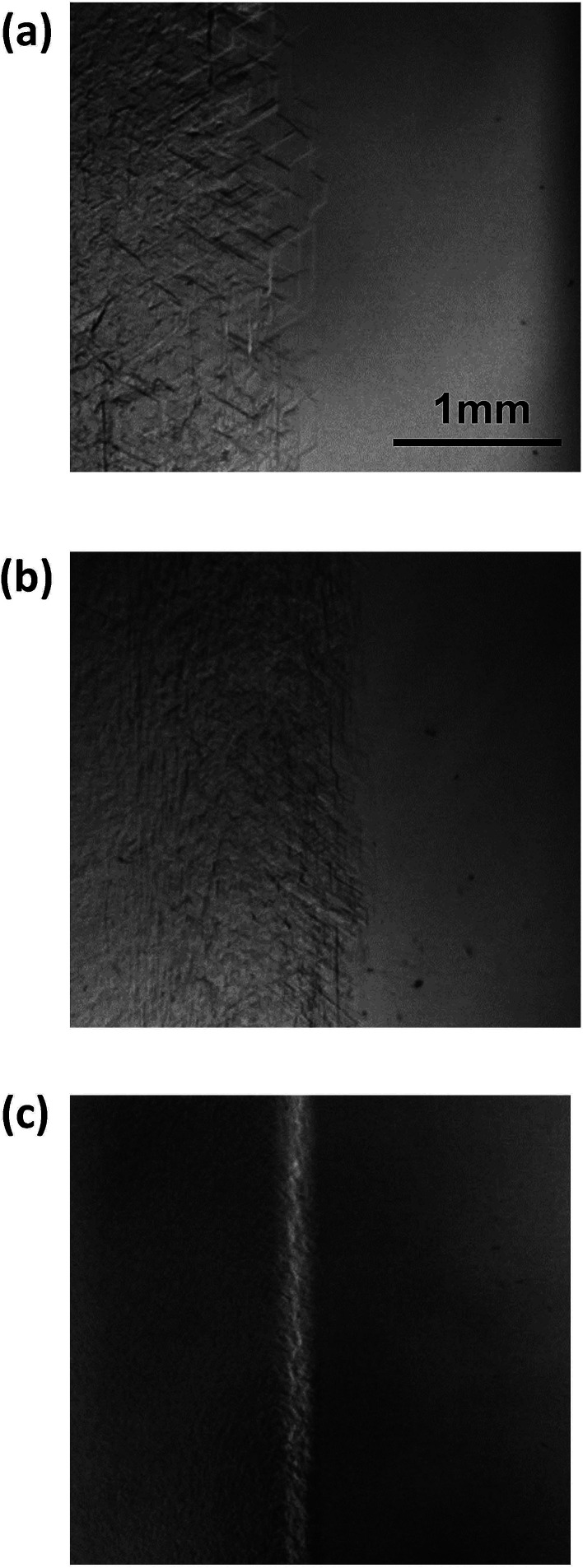
Steady state images of the hydrate growth interface with the additive Type I AFP at concentrations of *c* = (a) 0.05 wt%, (b) 0.2 wt% and (c) 0.5 wt% for *V* = 1 μm s^−1^.

**Fig. 9:**
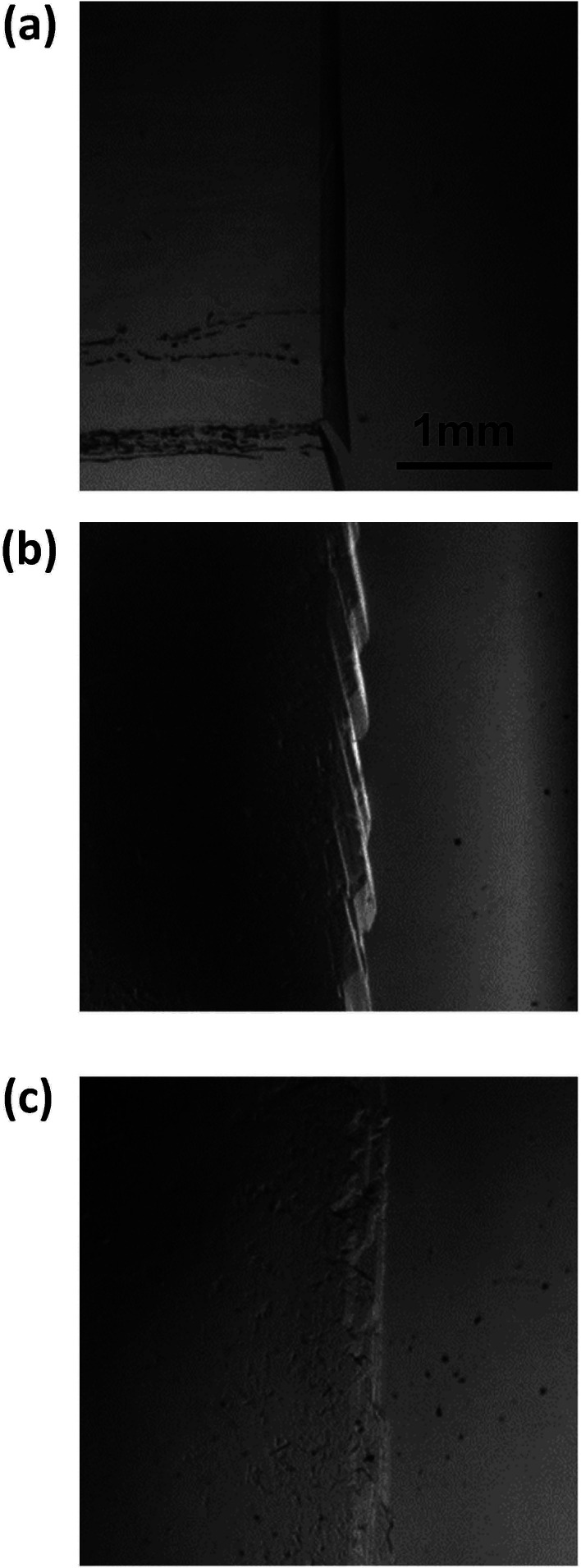
Steady state images of the hydrate growth interface with the additive Type III AFP at concentrations of *c* = (a) 0.05 wt%, (b) 0.2 wt% and (c) 0.5 wt% for *V* = 1 μm s^−1^.

**Fig. 10:**
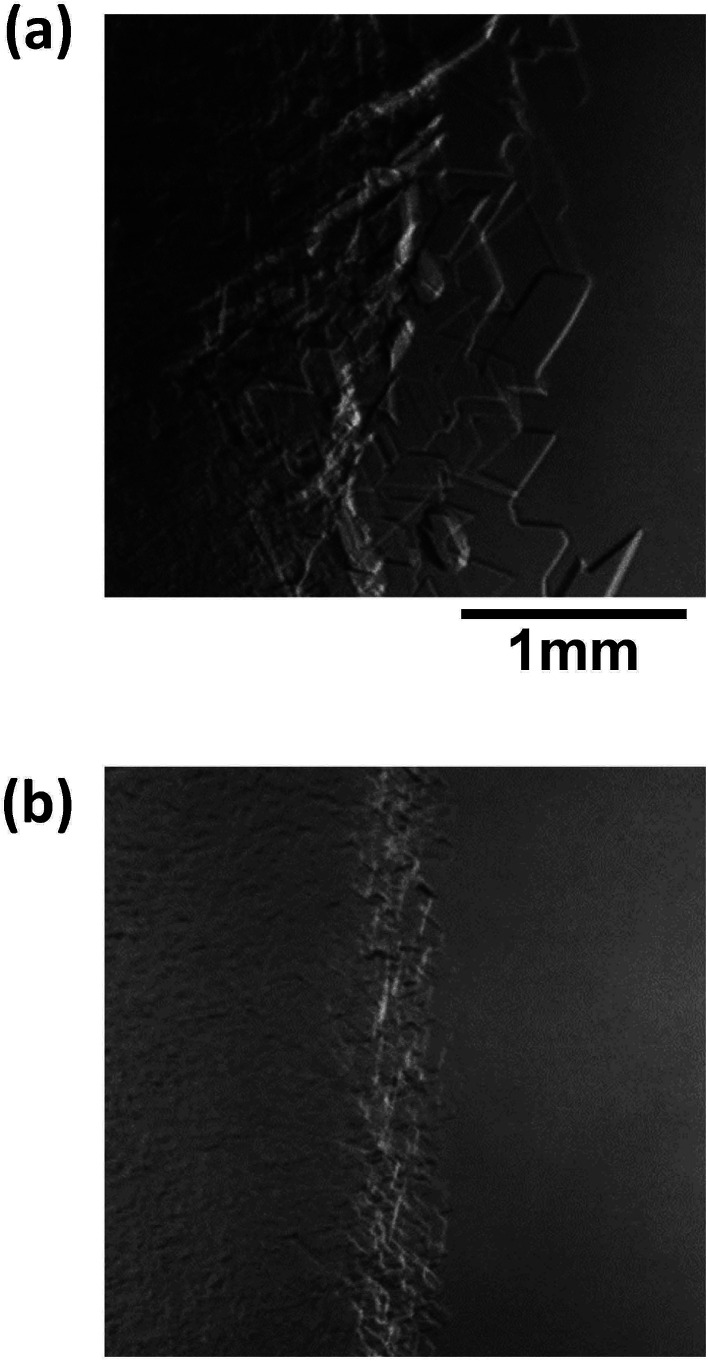
Steady state images of the hydrate growth interface with *V* = (a) 0.5 μm s^−1^ and (b) 10 μm s^−1^ for additive Type I AFP at a concentration of *c* = 0.05 wt%.

**Fig. 11:**
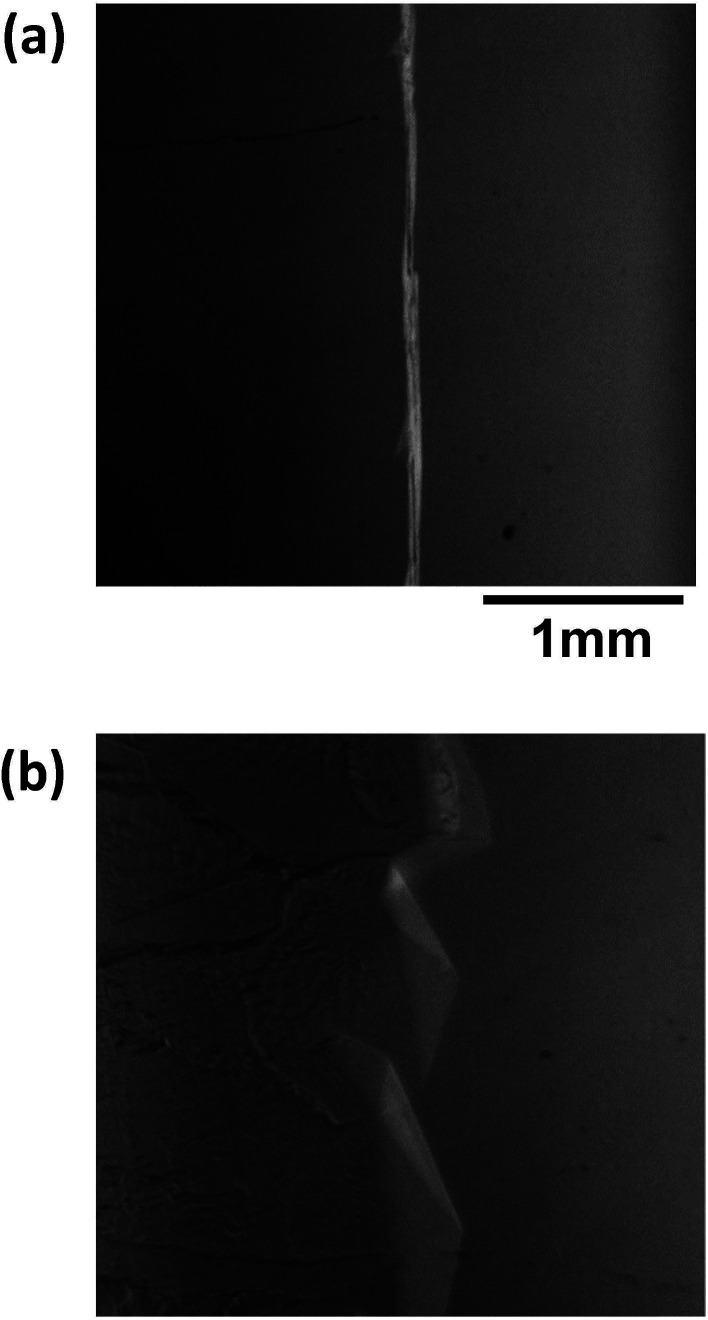
Steady state images of the hydrate growth interface for *V* = (a) 0.5 μm s^−1^ and (b) 10 μm s^−1^ for the additive Type III AFP with concentration of *c* = 0.05 wt%.

Fig. 8 shows images of the hydrate growth interface at steady state for different Type I AFP concentrations; *c* = (a) 0.05 wt%, (b) 0.2 wt% and (c) 0.5 wt% for *V* = 1 μm s^−1^. At *c* = 0.05 wt%, a hexagonal plate-like crystal forms across the whole growth interface. At *c* = 0.2 wt%, a relatively small hexagonal plate forms at the growth interface. At *c* = 0.5 wt%, the shape of the growth interface is relatively flat. Fig. 9 shows images of the hydrate growth interface at steady state for different concentrations of Type III AFP; *c* = (a) 0.05 wt%, (b) 0.2 wt% and (c) 0.5 wt% for *V* = 1 μm s^−1^. At *c* = 0.05 wt%, flat-plate crystals form. At *c* = 0.2 wt%, a concave–convex pattern appears at the interface, and at *c* = 0.5 wt%, many concave–convex pattern crystals are found at the growth interface. Fig. 10 shows images of the hydrate growth interface at steady state with *V* = (a) 0.5 μm s^−1^ and (b) 10 μm s^−1^ for added Type I AFP concentration *c* = 0.05 wt%. At *V* = 0.5 μm s^−1^, a flat-plate hexagonal crystal forms. At *V* = 10 μm s^−1^, the concave–convex pattern forms. Fig. 11 shows images of the hydrate growth interface at steady state with *V* = (a) 0.5 μm s^−1^ and (b) 10 μm s^−1^ for added Type III AFP concentration *c* = 0.05 wt%. At 0.5 μm s^−1^, the flat-plate crystal forms, and at *V* = 10 μm s^−1^, the concave–convex pattern forms.

Here, Type I AFP is more effective than Type III AFP. This seems to be related to a difference in how the AFP influences the hydrate crystal surface structure. Depending on its concentration, the presence of Type I AFP changes the crystal morphology drastically. Conversely, at all concentrations of Type III AFP, the same crystal morphology is found. This result suggests that crystal morphology is an important factor in determining KHI performance.

All KHIs in this study finely destabilized the growth interface. The many crystal boundary might entrap KHIs The flat Δ*T* dependence on *V* as shown in Fig. 7 likely results from such entrapping. The weak Δ*T* dependence on *V* is expected to lead to consistent on-site KHI performance.

## Conclusions

The kinetic inhibition performance of KHIs (Type I AFP, Type III AFP, PVA and PVP) for unidirectional THF hydrate crystal growth was evaluated. Supercooling at the growth interface was affected by the chemical species of the inhibitor and its concentration. The degree of supercooling caused by the AFP tends to be overestimated due to superheating, as also reported by Celik *et al.* Therefore, we measured the temperature of the growth interface directly using a thermocouple to avoid this overestimation.

The rank of kinetic inhibition performance of the studied compounds was found to be Type I AFP > PVP > Type III AFP > PVA.

Type I AFP changed the morphology of the THF hydrate crystal drastically depending on its concentration. Type III AFP did not show this effect. The difference in the effectiveness of these AFPs shows that their influence on the crystal morphology is an important factor for determining their antifreeze performance.

## Conflicts of interest

There are no conflicts to declare.

## Supplementary Material
